# Role of Savings and Internal Lending Communities (SILCs) in improving household wealth and financial preparedness for birth in rural Zambia

**DOI:** 10.1093/heapol/czab049

**Published:** 2021-04-28

**Authors:** HaEun Lee, Elisa M Maffioli, Philip T Veliz, Michelle L Munro-Kramer, Tenford K Phiri, Isaac Sakala, Jameson Kaunda, Nchimunya M Chiboola, Jody R Lori

**Affiliations:** Health Behavior and Biological Sciences, University of Michigan, School of Nursing, 400 North Ingalls Street, Ann Arbor, MI 48109-5482, USA; Health Management and Policy, University of Michigan, School of Public Health, 1415 Washington Heights, Ann Arbor, MI 48109, USA; Applied Biostatistics Laboratory, University of Michigan, School of Nursing, 400 North Ingalls Street, Ann Arbor, MI 48109-5482, USA; Health Behavior and Biological Sciences, University of Michigan, School of Nursing, 400 North Ingalls Street, Ann Arbor, MI 48109-5482, USA; Africare Zambia, Flat A, Plot 2407/10 MBX, Off Twin Palm Road, Ibex Hill, Lusaka 33921, Zambia; Africare Zambia, Flat A, Plot 2407/10 MBX, Off Twin Palm Road, Ibex Hill, Lusaka 33921, Zambia; Africare Zambia, Flat A, Plot 2407/10 MBX, Off Twin Palm Road, Ibex Hill, Lusaka 33921, Zambia; Africare Zambia, Flat A, Plot 2407/10 MBX, Off Twin Palm Road, Ibex Hill, Lusaka 33921, Zambia; Associate Dean of Global Affairs, Health Behavior and Biological Sciences, University of Michigan, School of Nursing, 400 North Ingalls Street, Ann Arbor, MI 48109-5482, USA

**Keywords:** Savings group, cost, financial barrier, maternal health, birth preparedness

## Abstract

Savings and Internal Lending Communities (SILCs) are a type of informal microfinance mechanism adapted in many low- and middle-income countries (LMICs) to improve financial resources for poor and rural communities. Although SILCs are often paired with other health and non-health-related interventions, few studies have examined SILCs in the context of maternal health. This study examined the association between SILC participation, household wealth and financial preparedness for birth. The study also examined the association between sex and financial preparedness for birth. A secondary analysis was conducted on individual survey data collected from SILC participants in two rural districts of Zambia between October 2017 and February 2018. A convenience sample of 600 participants (Lundazi: *n* = 297; Mansa: *n* = 303) was analysed. Descriptive analyses were run to examine SILC participation and household wealth. Multiple binary logistic regression models were fit to assess the unadjusted and adjusted relationship between (1) SILC participation and household wealth, (2) SILC participation and financial preparedness for birth and (3) sex and financial preparedness for birth. The results show that SILC participation led to an average increase of 7.32 items of the 13 household wealth items. SILC participants who had their most recent childbirth after joining SILCs were more likely to be financially prepared for birth [adjusted odds ratio (AOR): 2.99; 95% confidence interval (95% CI): 1.70-5.26; *P* < 0.001] than participants who had their most recent childbirth before joining SILCs. Females were more likely to be financially prepared for birth than males if they had their most recent birth before joining an SILC (AOR: 1.79; 95% CI: 1.16-2.66; *P* < 0.01). SILC participation is shown to increase household wealth and financial preparedness for birth for both men and women. SILCs are a promising intervention that can help poor and rural populations by increasing financial resources and financially preparing parents for birth.

**Key messages**

- Lack of financial resources is a significant barrier for women in low-income countries to deliver at a health facility.
- Savings and Internal Lending Community (SILC) is a type of microfinance mechanism that can improve household wealth for poor people living in rural areas.
- Participants who had (or their wife/partner had) the most recent delivery after joining an SILC had higher odds of being financially prepared for birth than participants who had their most recent delivery before joining an SILC.
- Females had higher odds of being financially prepared for birth than males among the participants who delivered their most recent child before joining SILCs.

## Introduction

Of the 295 000 maternal deaths that occur around the world every year, 99% occur in low-income countries and 66% in sub-Saharan Africa [Markos and Bogale, 2014; World Health Organization (WHO), 2019]. Nearly all maternal deaths can be prevented, as evidenced by the huge disparities found between the maternal death rates in high- and low-income countries (Nour, 2008; Obaid, 2007). Women in low-income countries face a disproportionately high burden of maternal deaths: the chance of a woman in a low-income country dying while giving birth is as high as 1 in 13, while the chance of a woman dying in a high-income country is 1 in 4100 (WHO, 2019). In Zambia, maternal deaths represent 10% of all deaths among women aged 15–49 years, with ∼252 maternal deaths per 100 000 live births (Central Statistical Office (CSO) Zambia, 2020).

There are numerous reasons why women in low- and middle-income countries (LMICs) are not seeking, reaching and receiving appropriate care in time (Black *et al.*, 2016). However, scholars agree the lack of financial resources is one of the greatest barriers to accessing fundamental reproductive health services such as antenatal care, postnatal care visits, family planning interventions and facility-based deliveries (Borghi *et al.*, 2006; Moyer and Mustafa, 2013; Sacks *et al.*, 2017). Therefore, women with fewer financial resources are more likely to bear the burden of preventable maternal deaths and mortality as compared with women with greater financial resources (Jennings *et al.*, 2017; Obaid, 2007).

While the lack of financial resources makes it challenging for rural and poor women in low-income countries to access facility-based delivery, sociocultural factors can exacerbate these issues. In many low-income countries in sub-Saharan Africa, spouses and older family members strongly influence a woman’s ability and decision to deliver at a health facility (Kalu-umeh *et al.*, 2013; Shaikh *et al.*, 2017). In most sub-Saharan African countries, males are still the primary income earners and the decision-makers of family finances (Sacks *et al.*, 2017; Sialubanje *et al.*, 2015; Tancred *et al.*, 2016). Therefore, women often rely on their husbands or partners to purchase required birth items and provide other necessary financial resources to prepare for birth (Sacks *et al.*, 2017; Sialubanje *et al.*, 2015; Tancred *et al.*, 2016).

A study examining household savings during pregnancy showed that 90% of the women who reported saving any money for their most recent birth had either husbands or parents contribute to their savings (Chiu *et al.*, 2019). Those who saved any money, compared with those who did not save for birth, were significantly more likely to deliver at a health facility (Chiu *et al.*, 2019). When male partners either fail or refuse to provide financial support, women are less likely to access facility-based delivery despite their personal desires (Tancred *et al.*, 2016). Alternatively, even when husbands or partners know and desire to support women to deliver at a health facility, they may not be able to because of limited or unstable income (Scott *et al.*, 2018; Tancred *et al.*, 2016). Therefore, innovative and culturally competent interventions financially empowering both women and their husband or partner to prepare for birth are critically needed.

Savings Groups (SGs)—low-risk, self-managed, self-financed and informal forms of microfinance—have been recognized for their ability to reach the extremely poor (those earning <1.90/day) in rural areas. They show great potential as a type of intervention that can financially empower both women and husbands or partners to prepare for birth (Parr and Bachey, 2015). There are many different models of SGs that have been developed and facilitated by over 70 organizations worldwide (Rippey *et al.*, 2015). SGs typically comprise 15–30 self-selected members who meet on a regular basis to contribute a set amount of funds within each member’s individual capacity (Karlan *et al.*, 2017; Ksoll *et al.*, 2016). The SG members can then access the accumulated savings as loans with low interest upon group approval. Once the SGs have operated for a predefined cycle, usually 9–12 months, the group members ‘share out’ the savings, loan repayments and interests accumulated over time (Karlan *et al.*, 2017).

Formed by community members, SGs also function as a social group for the participants to share ideas and stories during meetings, generating a sense of community (Taneja, 2013). Studies that have used SGs to enhance maternal health often include SGs not only as a financial intervention to help overcome financial barriers to access reproductive health services but also as a social platform for the women to discuss their reproductive health issues with each other to learn about the importance and availability of reproductive health services (Saggurti *et al.*, 2018; Shaikh *et al.*, 2017). However, studies generally focus on SGs as a social platform to deliver maternal and child health educational interventions rather than a financial mechanism to help overcome the financial barriers to accessing and utilizing reproductive health services (Lee *et al.*, 2020). Therefore, the present study aims to examine SGs as a financial mechanism to overcome financial barriers to safe delivery.

This study specifically examines the impact of the Savings and Internal Lending Communities (SILCs), an SG model developed by Catholic Relief Services, one of the most widely implemented models of SGs in Zambia (Ferguson, 2012; Taneja, 2013; Vanmeenen, 2006). Like other SGs, the SILCs primarily target women and provide a strategy to increase household income through self-managed and savings-led financial services (Ferguson, 2012; Taneja, 2013; Vanmeenen, 2006). Zambia’s SILCs not only target women but also men, allowing us to examine the SILCs’ impact on wealth and financial preparedness for birth, from the perspectives of both the participating women and their husbands or partners.

The purpose of the article is to examine the association between SILC participation, household wealth and financial preparedness for birth. Furthermore, the study also examined the association between sex and financial preparedness for birth. Financial preparedness for birth is often defined as saving money for birth-related expenses, assessed by asking the woman and/or her husband or partner whether she/he was able to purchase required supplies (e.g. baby clothes, gloves and plastic sheet) for the woman to deliver at health facilities (Chiu *et al.*, 2019; Tancred *et al.*, 2016). Because the types of supplies required may differ by health facilities, financial preparedness for birth was determined by the SILC participants subjectively answering whether they were able to purchase all the material they needed for the most recent delivery.

## Methods

### Overview

A secondary analysis was conducted on cross-sectional SILC impact survey data collected from SILC participants in two Zambian districts: Lundazi and Mansa. At the time of the SILC implementation and impact survey, data were collected from Lundazi and Mansa. Part of Mansa has now been split to make a new district (Chembe), but this change occurred after the implementation of the SILC and does not affect the results. The authors partnered with a local non-governmental organization (NGO) to implement SILCs as part of the Maternity Waiting Home (MWH) project in rural Zambia. The MWH project aimed to understand the impact of MWHs on reproductive health service access and maternal outcomes for women living far (>10 km) from health facilities (Lori *et al.*, 2018; Scott *et al.*, 2018). The local partner implemented the SILCs and collected the survey data. The selection process of 10 communities—5 from Lundazi and 5 from Mansa—where SILCs were implemented is the same as that of the MWH project (Lori *et al.*, 2018; Scott *et al.*, 2018). SILCs were first implemented in January 2016, and data were collected between October 2017 and February 2018 depending on how long the SILCs have been running.

### Sample

A convenience sample of 600 participants was sought from a total pool of 6711 participants from the 10 different communities SILC groups were implemented. Five of the communities resided are located in Lundazi (*n* = 297) and five in Mansa (*n* = 303).^1^ The local NGO’s programme evaluators met the groups on their monthly meeting dates. The description of the study was provided at the end of the regular SILC meetings, and the SILC members were asked to voluntarily participate in the survey. There were volunteers representing each of the 10 different communities. Volunteers for the survey provided verbal consent, and the survey was collected through in-person interviews in either English or the local dialect (e.g. Bemba, Nyanja and Tonga). The process was repeated for each SILC meeting until data reached 300 participants for each district. Inclusion criteria for participants were age 18 years or older and SILC group membership (must have participated for at least one cycle of committed timeline).

Ethical approvals for the MWH project were obtained from the authors’ Institutional Review Board (IRB), as well as from the ERES Converge Research IRB, a private local ethics board in Zambia.

### Measures

The purpose of the SILC impact survey was to understand how loan and share-out funds from SILCs were used, how the funds affected the members’ livelihood and how SILC members perceived SILCs. The SILC impact survey included three domains: (1) demographics, (2) economic outcomes and (3) non-economic outcomes and financial preparedness for birth.

#### Demographic domain

The demographic domain included information such as participant’s age, sex, district of residence, month and year of the participant’s most recent childbirth (for male participants we asked for their wife’s/partner’s most recent childbirth), and the month and year when they joined the SILC.

#### Economic domain

The economic domain included information on the amount of the first loan, usage of the loan and share-out funds, and engagement in agriculture, business and/or animal husbandry. Furthermore, data about the specific amounts of investments and gain from agricultural, business and animal husbandry before and after joining the SILC were gathered. The survey information regarding what materials comprised house and roofing structures before and after joining the SILC were also included. These questions were included in the economic domain because they are used to create a wealth index by many low-income countries’ Demographic and Health Surveys (Kolenikov and Angeles, 2009).

#### Non-economic domain

The non-economic domain included variables such as the ability to pay for child school fees, uniforms and shoes; food security and the ability to purchase all the required supplies for the most recent delivery. The survey ends with open-ended questions asking for examples of how membership in the SILC has helped the participant or their family, whether they would recommend SILC to their family and why they would or would not recommend the SILC membership.

#### Financial preparedness for birth

Last, the financial preparedness for birth was assessed by asking the participants whether he/she was able to purchase all the required supplies—plastic sheet, gloves, baby hat, baby clothes, wrap and so on—for the most recent delivery. The participants who answered ‘yes’ to the question were categorized as financially prepared birth and those who answered ‘no’ were categorized as not financially prepared for birth.

Because many people in low-income countries like Zambia often lack regular income, household wealth is frequently assessed by counting assets and assessing the quality of housing, sanitation facility and/or water supply (Kolenikov and Angeles, 2009). Similarly, to capture the impact of SILCs on household wealth, the ‘increase of wealth index’ variable was created using both the economic and non-economic variables.

Using these variables from the economic and non-economic domains, a total of 13 new discrete indicators were created. Each indicator was compared across two time points—before and after joining SILCs. Post-SILC participation improvements were coded as ‘1’. No change or post-SILC participation decline/decrease were coded as ‘0’. The ‘increase of wealth index’ was then created by summing the 13 new indicators. According to the United States Agency for International Development’s guideline for housing conditions (2016), brick and cement were considered improved housing materials. Metal and cement were considered improved roof materials. If participants reported having these improved materials for housing and/or roofing after joining the SILCs, the two variables were coded as ‘1’. The reliability coefficient for the increase of wealth index was 0.86 (0.8 > *α* ≥ 0.7 = acceptable; 0.9 > *α* ≥ 0.8 = good and *α* ≥ 0.9 = excellent).

To understand the impact of SILC participation on financial preparedness for birth, all SILC participants were divided into two groups: those who had (or their wife/partner had) their most recent childbirth before joining an SILC and those who had (or their wife/partner had) their most recent childbirth after joining an SILC. The sample was dichotomized by the most recent childbirth date and SILC initial join date to assess how income earned through SILCs influence financial preparedness for birth.

### Analysis

The aim of this analysis was to describe SILC participation and household wealth for birth and to examine the association between (1) increase of wealth and financial preparedness for birth and (2) sex of the participants and financial preparedness for birth. Descriptive statistics were analysed with means and standard deviations (SD) provided for the overall sample as well as the stratified sample between those who were financially prepared for birth and those who were not. A set of chi-square tests of independence and two sample *t*-tests were conducted to examine the differences between participants who were financially prepared and participants who were not for the overall and stratified samples. The financially prepared sample was further stratified by sex.

Means and SD were calculated for the overall and stratified samples from Lundazi and Mansa. Several binary logistic regression models were fit to assess the unadjusted and adjusted relationship between increased wealth index and financial preparedness for birth. Adjusted logistic regression models included age, sex, district of residence and the period of the most recent childbirth as covariates. All logistic regression models provided adjusted odds ratios (AORs) and 95% confidence intervals (95% CIs).

To understand the relationship between sex and financial preparedness for birth, logistic regression models were fit between those who had their most recent childbirth before and after joining SILCs. The data were analysed using Stata 15.0 (StataCorp, College Station, TX, USA).

## Results

### Sample characteristics

Table 1 presents descriptive statistics for the total sample of 600 SILC participants (Lundazi: 297; Mansa: 303). Approximately half of the sample resided in Lundazi (49.50%) and the other half in Mansa (50.50%). More females (64.67%; *n* = 388) than males were included in the sample. Approximately 30% of the sample was between 26 and 35 years of age (*n* = 183), closely followed by 36-year-olds to 45-year-olds (28.55%; *n* = 171). About one-fifth (19.88%) of the overall sample had their most recent childbirth after joining SILCs. On average, the increase of wealth index was 7.32, which means that, on average, SILC participants had ∼7.32 of the 13 economic and noneconomic indicator increase after SILC participation.

**Table 1.** Descriptive statistics for overall demographics and stratified sample of participants who identified as financially prepared for birth

	Overall	Not financially prepared for birth	Financially prepared for birth	*P*-value
Total, *n* (%)	600	187 (35.15)	345 (64.85)	
District, *n* (%)				<0.0001***
Lundazi	297 (49.50)	127 (67.91)	169 (48.99)	
Mansa	303 (50.50)	60 (32.09)	176 (51.01)	
Sex, *n* (%)				0.009**
Male	212 (35.33)	81(43.32)	110 (31.88)	
Female	388 (64.67)	106 (56.68)	235 (68.12)	
Age (years), *n* (%)				0.552
18–25	138 (23.04)	41 (22.04)	81 (23.48)	
26–35	183 (30.55)	60 (32.26)	108 (31.30)	
36–45	171 (28.55)	51(27.42)	104 (30.14)	
>46–55	107 (17.86)	34 (18.28)	52 (15.07)	
Child born period, *n* (%)				0.001**
Before joining an SILC	411 (80.12)	144 (87.80)	230 (74.43)	
After joining an SILC	102 (19.88)	20 (12.20)	79 (25.57)	
Increase of wealth index, mean (SD)	7.32 (3.77)	8.52 (3.61)	7.54 (3.44)	0.002**
Lundazi	10.55 (1.76)	10.71 (1.56)	10.42 (1.89)	0.161
Mansa	4.15 (2.20)	3.88 (1.95)	4.77 (2.04)	0.003**
Increased after joining SILC, *n* (%)				
Business	450 (75.00)	155 (82.89)	272 (78.84)	0.263
Food	301 (50.17)	126 (67.38)	156 (45.22)	<0.001 ***
Roof material improved	187 (31.17)	73 (39.04)	113 (32.75)	0.147
Home material improved	198 (33.00)	70 (37.43)	125 (36.23)	0.784
Land	489 (81.50)	151 (80.75)	301 (87.25)	0.045*
Seed bought	436 (72.67)	144 (77.01)	264 (76.52)	0.900
Fertilizer bought	424 (70.67)	144 (77.01)	259 (75.07)	0.619
Harvest amount	460 (76.67)	148 (79.14)	286 (82.90)	0.286
Livestocks	409 (68.17)	142 (75.94)	253 (73.33)	0.512
New bicycle	247 (41.17)	111(59.36)	129 (37.39)	<0.001***
Uniform for children	250 (41.67)	94 (50.27)	150 (43.48)	0.134
School fee for children	248 (41.33)	109 (58.29)	134 (38.84)	<0.001***
Shoes for children	293 (48.83)	127 (67.91)	160 (46.38)	<0.001***

Chi-square tests and two sample *t*-tests were conducted to examine the difference between participants who were financially prepared and participants who were not.

* *P* < 0.05;

** *P* < 0.01;

*** *P* < 0.001.

Of the 600 participants, 64.85% were considered financially prepared for the most recent birth. When comparing the two groups—financially prepared for birth and not financially prepared for birth—the result showed a significant difference in the district of residence (*P* < 0.001), sex distribution (*P* = 0.009), the time point at which the most recent childbirth occurred (*P* < 0.001) and increase in wealth (*P* = 0.002). Samples that were financially prepared for the most recent birth had a higher percentage of participants from Mansa (51.01%) compared with 32.09% of those not financially prepared. The financially prepared sample also had more female (68.12 %) and more participants delivering a child after joining SILCs (25.57%) compared with 56.68% female and 12.20% participants delivering after joining SILCs from the not financially prepared sample. The financially prepared sample had, however, a lower increase of wealth, with an average increase of 7.54 wealth index compared with 8.52 from the not financially prepared. While we find no evidence that increase of wealth is significantly different between financially prepared and not financially prepared SILC participants from Lundazi, a large gap existed in the increase of wealth index between the two districts, with participants from Lundazi (10.49) exhibiting a greater increase of wealth index in the sample that were financially prepared compared with those from Mansa (2.04).

Table 2 shows the association between demographic variables, increase of wealth and 13 economic and non-economic indicators and financial preparedness for birth. Participants from Mansa presented significantly higher odds of being financially prepared (AOR: 3.15; 95% CI: 1.41-7.03) than participants from Lundazi. Furthermore, females (AOR: 1.76; 95% CI: 1.16-2.66) compared with males, and those who had their most recent delivery after joining SILCs (AOR: 2.99; 95% CI: 1.70-5.26) compared with those who had their most recent delivery before joining SILCs, showed greater odds of being financially prepared for birth. The association between the increase of wealth and financial preparedness for birth was statistically significant for the participants from Mansa (AOR: 1.26; 95% CI: 1.03-1.55) but not for the participants from Lundazi (AOR: 0.90 95% CI: 0.79-1.04).

**Table 2.** Predictors of financial preparedness for the overall sample

	Financial preparedness for birth	Financial preparedness for birth OR (95% CI)	Financial preparedness for birth AOR (95% CI)
Total, *n*/*N*	345/600		473/600
District, *n* (%)			
Lundazi	169 (48.99)	Reference	Reference
Mansa	176 (51.01)	2.20 (1.51–3.19)***	3.15 (1.41–7.03)**
Sex, *n* (%)			
Male	110 (31.88)	Reference	Reference
Female	235 (68.12)	1.63 (1.13–2.35)**	1.76 (1.16–2.66)**
Age mean (SD)	35.02 (10.42)	0.99 (0.97–1.01)	1.00 (0.98–1.02)
Child born period, *n* (%)			
Before joining SILCs	230 (74.43)	Reference	Reference
After joining SILCs	79 (25.57)	2.47 (1.45–4.21)**	2.99 (1.70–5.26)***
Increase of wealth index mean (SD)	7.54 (3.44)	0.92 (0.87–0.97)**	1.01 (0.90–1.13)
Lundazi	10.42 (1.89)	0.90 (0.79–1.03)	0.90 (0.79–1.04)
Mansa	4.77 (2.04)	1.24 (1.07–1.45)**	1.26 (1.03–1.55)*
Increased after joining SILC, *n* (%)			
Business	272 (78.84)	0.76 (0.48–1.21)	1.33 (0.75–2.36)
Food	156 (45.22)	0.39 (0.27–0.57)***	0.55 (0.33–0.92)*
Roof material improved	113 (32.75)	0.76 (0.52–1.10)	1.02 (0.62–1.68)
Home material improved	125 (36.23)	0.94 (0.65–1.37)	1.51 (0.91–2.50)
Land	301 (87.25)	1.63 (1.00–2.64)*	2.88 (1.41–5.91)**
Seed bought	264 (76.52)	0.97 (0.63–1.48)	1.32 (0.70–2.50)
Fertilizer bought	259 (75.07)	0.89 (0.59–1.36)	2.00 (0.96–4.13)
Harvest amount	286 (82.90)	1.27 (0.81–2.00)	2.49 (1.21–5.11)*
Livestock	253 (73.33)	0.87 (0.57–1.31)	1.52 (0.81–2.84)
New bicycle	129 (37.39)	0.40 (0.28–0.58)***	0.46 (0.26–0.82)**
Uniform for children	150 (43.48)	0.76 (0.53–1.08)	1.03 (0.60–1.75)
School fee for children	134 (38.84)	0.45 (0.31–0.65)***	0.54 (0.32–0.90)*
Shoes for children	160 (46.38)	0.40 (0.28–0.59)***	0.38 (0.21–0.70)**

Adjusted model accounted for participants’ sex, age, district, time of the most recent birth and increase of wealth, but the 13 individual wealth variables were not part of the adjusted model.

SD: standard deviation; SILC: Savings and Internal Lending Community; OR: odds ratio; CI: confidence interval; AOR: adjusted odds ratio.

* *P* < 0.05;

** *P* < 0.01;

*** *P* < 0.001.

Tables 3 and 4 examine the association between demographic variables, increase of wealth, the 13 economic and non-economic indicators and financial preparedness for birth. The participant or the participant’s wife/partner having the most recent childbirth after joining an SILC increased the odds of financial preparedness when compared with participants who had the most recent childbirth before joining an SILC; this was found for both participants from Lundazi (AOR: 2.42; 95% CI: 1.31-4.47) and Mansa (AOR: 10.15; 95% CI: 1.28-80.12). In addition, an increase in the wealth index was shown to be significantly associated with an increase in the odds of indicating financial preparedness for birth in Mansa only (AOR: 1.26; 95% CI: 1.03-1.55).

**Table 3.** Predictors of financial preparedness for the Lundazi sample

	Financial preparedness for birth (Lundazi)	Financial preparedness for birth OR (95% CI)	Financial preparedness for birth AOR (95% CI)
Total, *n*/*N*	169/297		293/297
Sex, *n* (%)			
Male	52 (30.77)	Reference	Reference
Female	117 (69.23)	1.56 (0.96–2.52)	1.79 (1.08–2.97)*
Age mean (SD)	33.01 (8.41)	0.99 (0.96–1.01)	1.00 (0.97–1.03)
Child born period, *n* (%)			
Before joining SILCs	120 (71.01)	Reference	Reference
After joining SILCs	49 (28.99)	2.25 (1.24–4.07)**	2.42 (1.31–4.47)**
Increase of wealth index mean (SD)	10.42 (1.89)	0.90 (0.79–1.03)	0.90 (0.79–1.04)
Increased after joining SILC, *n* (%)			
Business	147 (86.98)	0.69 (0.33–1.46)	0.85 (0.38–1.91)
Food	128 (75.74)	0.80 (0.46–1.40)	0.91 (0.48–1.73)
Roof material improved	97 (57.40)	1.16 (0.73–1.85)	1.56 (0.88–2.76)
Home material improved	105 (62.13)	1.46 (0.92–2.34)	2.11 (1.18–3.77)*
Land	163 (96.45)	1.11 (0.33–3.73)	1.66 (0.46–5.96)
Seed bought	153 (90.53)	0.07 (0.00–0.58)*	0.08 (0.01–0.65*
Fertilizer bought	166 (98.22)	1.33 (0.26–6.74)	1.38 (0.25–7.42)
Harvest amount	162 (95.86)	0.18 (0.02–1.51)	0.20 (0.02–1.72)
Livestock	157 (92.90)	0.42 (0.13–1.35)	0.43 (0.13–1.43)
New bicycle	118 (69.82)	0.45 (0.25–0.81)**	0.47 (0.24–0.91)*
Uniform for children	123 (72.78)	1.41 (0.86–2.33)	2.15 (1.12–4.13)*
School fee for children	114 (67.46)	1.02 (0.62–1.67)	1.21 (0.65–2.27)
Shoes for children	129 (76.33)	0.33 (0.16–0.67)**	0.30 (0.12–0.73)**

Adjusted model accounted for participants’ sex, age, district, time of the most recent birth and increase of wealth, but the 13 individual wealth variables were not part of the adjusted model.

SD: standard deviation; SILC: Savings and Internal Lending Community; OR: odds ratio; CI: confidence interval; AOR: adjusted odds ratio.

* *P* < 0.05;

** *P* < 0.01;

**Table 4.** Predictors of financial preparedness for Mansa sample

	Financial preparedness for birth (Mansa)	Financial preparedness for birth OR (95% CI)	Financial preparedness for birth AOR (95% CI)
Total, *n*/*N*	176/303		180/303
Sex, *n* (%)			
Male	58 (32.95)	Reference	Reference
Female	118 (67.05)	1.90 (1.04–3.45)*	1.60 (0.76–3.39)
Age mean (SD)	36.96 (11.74)	0.98 (0.95–1.00)	0.99 (0.96–1.02)
Child born period, *n* (%)			
Before joining SILCs	110 (78.57)	Reference	Reference
After joining SILCs	30 (21.43)	10.63 (1.40–80.62)*	10.15 (1.28–80.12)*
Increase of wealth index mean (SD)	4.77 (2.04)	1.24 (1.07–1.45)**	1.26 (1.03–1.55)*
Increased after joining SILC, *n* (%)			
Business	125 (71.02)	1.22 (0.65–2.29)	1.57 (0.64–3.80)
Food	28 (15.91)	0.26 (0.13–0.50)***	0.28 (0.11–0.68)**
Roof material improved	16 (9.09)	1.09 (0.38–3.14)	0.36 (0.10–1.27)
Home material improved	20 (11.36)	2.43 (0.69–8.50)	0.91 (0.22–3.72)
Land	138 (78.41)	3.88 (2.08–7.22)***	2.63 (1.00–6.92)*
Seed bought	111 (63.07)	3.98 (2.11–7.49)***	3.31 (1.32–8.30)*
Fertilizer bought	93 (52.84)	2.24 (1.21–4.13)*	1.40 (0.52–3.75)
Harvest amount	124 (70.45)	4.11 (2.22–7.63)***	4.72 (1.69–13.18)**
Livestock	96 (54.55)	2.58 (1.39–4.81)**	2.44 (1.04–5.72)*
New bicycle	11 (6.25)	0.73 (0.24–2.20)	1.01 (0.18–5.67)
Uniform for children	27 (15.34)	0.80 (0.37–1.74)	0.25 (0.09–0.71)**
School fee for children	20 (11.36)	0.19 (0.09–0.38)***	0.13 (0.04–0.35)***
Shoes for children	31 (17.61)	0.85 (0.40–1.79)	0.53 (0.20–1.39)

Adjusted model accounted for participants’ sex, age, district, time of the most recent birth and increase of wealth, but the 13 individual wealth variables were not part of the adjusted model.

SD: standard deviation; SILC: Savings and Internal Lending Community; OR: odds ratio; CI: confidence interval; AOR: adjusted odds ratio.

* *P* < 0.05;

** *P* < 0.01;

*** *P* < 0.001.

In Table 5, sex of the participants was used to predict the odds of being financially prepared for the most recent birth. Females had greater odds of reporting being financially prepared for birth (AOR: 1.68; 95% CI: 1.07-2.65) than males for those who had their most recent childbirth before joining SILCs. For the participants who had their most recent childbirth after joining SILCs, sex had no statistically significant association with financial preparedness for birth.

**Table 5.** Sex predicting financial preparedness for birth between participants who had their most recent delivery before joining an SILC and participants who had their most recent delivery after joining an SILC

	Financial preparedness for birth AOR (95% CI)	
	Child born before joining SILC	Child born after joining SILC
Sex		
Male	Reference	Reference
Female	1.68 (1.07–2.65)*	2.04 (0.69–6.01)

SILC: Savings and Internal Lending Community; CI: confidence interval; AOR: adjusted odds ratio; adjusted model accounted for participants’ age, district and increase of wealth.

* *P* < 0.05.

## Discussion

Overall, the results show that participating in SILCs led to increased household wealth (indicated by individual wealth indicators and increased wealth index) and financial preparedness for birth. Furthermore, being a female was positively associated with financial preparedness for birth only if they had their most recent birth before joining an SILC.

### SILC participation and household wealth

The results show that SILC participation was positively associated with household wealth as evident in the increase of wealth index (average 7.32). This finding is congruent with the general literature, which suggests SGs are able to reach poor people living in rural areas to provide them the means to access basic financial services such as loans, social funds and share-out funds (Hermes and Lensink, 2011; Karlan *et al.*, 2017). This financial revenue allows them to invest in business, purchase land and livestock, pay for children’s school and purchase food (Hermes and Lensink, 2011; Parr and Bachey, 2015). However, it is important to note that the data do not show the amount of increase for each of the economic and non-economic indicators because participants did not use a standardized unit to report the increase. Hence, the data were not comparable across the participants. A cluster randomized control trial conducted in Malawi found that the SGs were able to reach some of the poorest households and could improve food security, housing standards and household assets and increase the number of economic activities and savings. However, there were no significant changes in the total income generated through economic activities (Ksoll *et al.*, 2016). Another randomized control trial examining the effect of SGs in Mali over 3 years showed positive but small effects in overall savings, amounts of money borrowed, households’ livestock holdings and food security [Innovations for Poverty Action (IPA), Bureau of Applied Research in Anthropology (BARA), University of Arizona, 2013]. Once again, there were no significant differences found on assessing for various savings, health expenses, school enrolment, business development or expansion and agricultural assets [Innovations for Poverty Action (IPA), Bureau of Applied Research in Anthropology (BARA), University of Arizona, 2013]. Although the present results show SILC participation and wealth increase were positively associated, we cannot measure the magnitude of increase for each of the 13 wealth indicators with the available data.

### SILC participation and financial preparedness for birth

Compared with the participants who had their most recent childbirth before joining SILCs and those who had their or their wife/partner had the most recent childbirth after joining SILCs were almost three times more likely to be financially prepared for birth, determined by the participants’ ability to purchase all the required supplies for the most recent delivery. Out-of-pocket costs relating to childbirth can range up to one-third of the monthly household income for the poorest Zambian households (Kaiser *et al.*, 2019). Poor families in rural areas are even more financially vulnerable during pregnancy and childbirth because they have limited access to cash and live farther away from health facilities (Borghi *et al.*, 2006). A study conducted in seven rural districts of Zambia—including Lundazi and Mansa—showed that baby clothes/blankets, delivery supplies such as disinfectant or cord clamps, and transportation were the most common expenditure related to delivery (Kaiser *et al.*, 2019). Furthermore, the study showed the average spending per childbirth was ∼$28.760 USD, calling attention to programmes that can help alleviate these expenses to increase accessibility to facility-based delivery (Kaiser *et al.*, 2019). The positive association between SILC participation and financial preparedness for birth shows the potential of SILCs as an innovative solution to overcome financial barriers related to childbirth.

The positive association between giving birth after joining SILCs and financial preparedness for birth was mostly replicated in the stratified analyses between Lundazi and Mansa. The likelihood of being financially prepared for birth was ∼2.5 times higher for those whose most recent childbirth occurred after joining an SILC in Lundazi and 10 times higher in Mansa. Furthermore, the increase in the wealth index was significantly associated (Tables 2 and 3) with financial preparedness for birth only in the Mansa sample. However, as wealth increased for Lundazi sample, the odds of being financially prepared for birth increased. These differences between the districts may be due to the difference in rurality, which may suggest a difference in education level, a covariate frequently shown to predict birth preparedness (Markos and Bogale, 2014). According to 2018 statistics, the median number of school years completed among Zambian males is 6.9 years and that among Zambian females is 6.8 years [Central Statistical Office (CSO) Zambia, 2020]. Not surprisingly, a large difference in schooling years, 2.7 years for females and 1.7 years for males, exists between Zambians living in rural and urban areas (Central Statistical Office (CSO) Zambia, 2020). Therefore, the Mansa sample may have a higher level of education on average, which may then influence financial resource prioritization for birth preparedness. Unfortunately, the SILC impact survey data did not include information such as education level to support these speculations.

### Sex and financial preparedness for birth

Overall, females were more likely to be financially prepared for birth than males for participants who had their most recent childbirth before joining SILCs. Effect of sex on financial preparedness for birth was not significant for participants who had their most recent childbirth after joining SILCs. One potential explanation maybe the function of SILCs as a social platform. SGs like SILC are shown to be conducive platforms for participants to discuss various issues, which develops trust, solidarity, collective efficacy and a sense of belonging within the group (Saha *et al.*, 2013; 2015; Lee *et al.*, 2020). Studies have shown the participating in SGs not only led to financial autonomy for females but also increased male’s participation in preparing for birth (Shaikh *et al.*, 2017; Ekirapa-Kiracho *et al.*, 2016; Lee *et al.*, 2020). Therefore, females sharing their concerns and difficulties regarding pregnancy and childbirth may have increased males' awareness regarding the importance of financial preparedness for birth. (Ekirapa-Kiracho *et al.*, 2016; Lee *et al.*, 2020).

When the sample was stratified, between those who had their most recent delivery before joining SILCs and those who had their most recent delivery after joining SILCs, the odds of females indicating being financially prepared for birth compared were 68% times higher when compared with males. For SILC participants who had their most recent delivery after joining SILCs, sex was no longer associated with financial preparedness for birth. While SILCs were not directly paired with specific educational interventions, SGs have been shown to provide important platforms for community members to network, interact and share various life events with each other (Shaikh *et al.*, 2017). During regular SILC meetings, participants can share information about personal life events such as pregnancy and childbirth. This experience can then inform males about the decision to prioritize financial resources for preparing for birth. It is well established that male’s knowledge and involvement with maternal and child health are directly associated with improved utilization of reproductive health services and maternal health outcomes (Yargawa and Leonardi-Bee, 2015). While male involvement is gradually improving in many sub-Saharan African countries, sex structures in society and cultural beliefs that pregnancy and childbirth are solely females’ responsibility still prevent males from gaining increased knowledge on pregnancy and childbirth (Dudgeon and Inhorn, 2004). Previous studies that paired SGs with maternal health education interventions have shown that participating in SGs leads to increased health knowledge and awareness of services not only for females but also for other participating community members. As such, females who participated in SGs were able to practice better health behaviours due to the increased knowledge, awareness and involvement among males and other community members who had also participated in the SGs (Ekirapa-Kiracho *et al.*, 2017). Therefore, a social platform where both males and females can converse about various issues, including pregnancy and childbirth, could have better informed males about the importance of preparing financially for birth.

## Limitations

While there were many strengths in the current study, several limitations need to be addressed. First, because the SILC impact survey was conducted cross-sectionally at the end of the cycle, ∼9–12 months since the beginning of the SILC cycle, it is subject to recall bias. Questions asked retrospectively on the investments and gain from agriculture, business and animal husbandry in relation to two different time points (before and after joining SILCs) are especially prone. Social desirability could have also impacted the outcomes, given that the survey data were collected through face-to-face interview with the local NGO’s programme evaluators rather than anonymously filled by participants. Thus, the participants may have overreported on the gain from the SILCs. However, the interview format was unavoidable due to the overall limited literacy in rural Zambia.

Second, the increase of wealth index is limited in its ability to capture wealth. Since responses were not recorded according to a specific unit for the investments and gain from agriculture, business and animal husbandry, the answers varied across participants. Some answered in Kwacha, while others responded in number of bags, kilograms, gallons and other units for indicators such as amount of crops harvested before and after joining SILCs. Therefore, while the study showed the overall increase of wealth in all SILC participants, it is unclear what the magnitude of increase was for each variable. Moreover, the survey did not capture other information that could have also shown an increase such as healthcare expenses. However, for many people in Zambia that lack regular income, assessing household wealth via counting the assets and quality of housing and water supply is a common methodology (Kolenikov and Angeles, 2009).

Last, the lack of additional variables such as expenditure on different birth supplies, transportation, drugs and diagnostic tests was not included in the survey. These variables would have provided deeper insights to assess the range of financial preparedness for birth. Because the type of supplies needed to deliver at a health facility may differ by health facility or woman (e.g. if a woman lives closer to a health facility, she does not need to prepare transportation fee), financial preparedness for birth was captured by asking the participants to report their perception whether they were able to purchase ‘all’ the necessary birth supplies. This may have caused the participants who were only partially prepared for birth to either overreport or underreport their financial preparedness for birth.

## Conclusion

The study found that participating in SILCs increased household wealth and the likelihood to be financially prepared for birth. In addition, female SILC participants were more likely to be financially prepared for birth only for the participants who had their most recent childbirth before joining SILCs. This finding suggests that SILCs may be functioning as a social platform for females to share their concerns regarding childbirth, which allowed both males and females to prioritize gains from SILCs to financially prepare for birth. In sum, the study suggests that SILCs are a promising intervention not only to increase wealth for the poor and rural populations but also to help participants be financially prepared for birth. As such, the present study holds important implications for improving maternal health by helping poor males and females living in rural areas to overcome financial barriers to access fundamental reproductive health services.

## Data Availability

The data underlying this article will be shared on reasonable request to the corresponding author.

## Endnotes

1. While the original study was not purposefully powered to address the specific research question, the current sample was sufficient to address the study aims, namely, the sample included 513 respondents who had a child during the study period, 411 (80.1%) had a child before joining an SILC and 102 (19.9%) had a child after joining an SILC. Using financial preparedness at time of birth as an example, 56% (*n* = 230) of respondents (those who had a child before joining an SILC) indicated being financially prepared when their child was born, while 77% (*n* = 79) of respondents (those who had a child after joining an SILC) indicated being financially prepared when their child was born. In order to detect a difference of this magnitude (with respect to financial preparedness) between respondents who had a child born before or after joining an SILC (i.e. OR = 2.60) at a 0.01 significance level with 90% power, the anticipated minimum effective sample size needed would be 170 respondents (allowing for inclusion of covariates that explain up to 20% of the variance). Given the current sample size, we have sufficient power to detect these differences in both the full sample and when stratified between the two districts.
